# Analgesic efficacy of collagen peptide in knee osteoarthritis: a meta-analysis of randomized controlled trials

**DOI:** 10.1186/s13018-023-04182-w

**Published:** 2023-09-16

**Authors:** Chun-Ru Lin, Sung Huang Laurent Tsai, Ko-Yen Huang, Po-An Tsai, Hsuan Chou, Shu-Hao Chang

**Affiliations:** 1 Department of Medical Education, Chang Gung Memorial Hospital, Linkou Branch, No. 5, Fuxing Street, Guishan District, Taoyuan City 333, Taiwan; 2 Department of Orthopaedic Surgery, Chang Gung Memorial Hospital, Keelung Branch, and Chang Gung University, F7, No 222 Mai-King Road, Keelung, Taiwan; 3 Department of Medical Education, Taipei Medical University-Shuang Ho Hospital, No. 291, Zhongzheng Road, Zhonghe District, New Taipei City, 235041 Taiwan; 4 School of Medicine, College of Medicine, Fu Jen Catholic University, New Taipei City, 242008 Taiwan; 5 Department of Orthopedics, Fu Jen Catholic University Hospital, Fu Jen Catholic University, No. 69, Guizi Rd., Taishan Dist., New Taipei City, 24352 Taiwan; 6 School of Medicine, College of Medicine, Fu Jen Catholic University, No. 510, Zhongzheng Rd., Xinzhuang Dist., New Taipei City, 24205 Taiwan

**Keywords:** Osteoarthritis, Knee osteoarthritis, Collagen peptide, Pain, Adverse events

## Abstract

**Background:**

The management of knee osteoarthritis involves various treatment strategies. It is important to explore alternative therapies that are both safe and effective. Collagen peptides have emerged as a potential intervention for knee osteoarthritis. This study aims to evaluate the analgesic effects and safety of collagen peptide in patients diagnosed with knee osteoarthritis.

**Methods:**

We conducted a systematic literature search following the guidelines of the Preferred Reporting Items for Systematic Reviews and Meta-Analyses statement. Multiple databases including PubMed, Scopus, EMBASE, Web of Science, Cochrane, and ClinicalTrials.gov were searched for randomized controlled trials (RCTs) published up to 27 May 2023 that focused on the analgesic outcomes and adverse events associated with collagen peptides or hydrolyzed collagen in patients with osteoarthritis. We assessed the quality of the included studies and the strength of evidence using the Cochrane ROB 2.0 tool and Grading of Recommendations, Assessment, Development, and Evaluations.

**Results:**

Four trials involving 507 patients with knee osteoarthritis were included and analyzed using the random-effects model. All these trials were considered to have a high risk of bias. Our results revealed a significant difference in pain relief between the collagen peptide group and the placebo group in patients with knee osteoarthritis (standardized mean difference: − 0.58; 95% CI − 0.98, − 0.18, p = 0.004; I2: 68%; quality of evidence: moderate). However, there was no significant difference in the risk of adverse events between collagen peptide and placebo (odds ratio: 1.66; 95% CI 0.99, 2.78, p = 0.05; I2: 0%; quality of evidence: very low).

**Conclusions:**

Our findings demonstrate significant pain relief in patients with knee osteoarthritis who received collagen peptides compared to those who received placebo. In addition, the risk of adverse events did not differ significantly between the collagen peptide group and the placebo group. However, due to potential biases and limitations, well-designed randomized controlled trials are needed to validate and confirm these findings.

**Supplementary Information:**

The online version contains supplementary material available at 10.1186/s13018-023-04182-w.

## Introduction

Knee osteoarthritis, a chronic degenerative joint disorder, can have a significant impact on the lives of those affected [1]. The condition involves gradual degradation of knee cartilage, accompanied by development of osteophytes and remodeling of the subchondral bone. This leads to chronic pain, limited mobility, and decreased mental well-being, greatly affecting the overall quality of life for individuals with knee osteoarthritis [2]. Various treatment options are available, ranging from lifestyle modifications and oral medications to intra-articular injections and surgical interventions in severe cases [1]. Factors such as age, presence of infrapatellar synovitis, comorbidities, ethnicity, body mass index, joint effusion, and severity of knee osteoarthritis at baseline can influence the prognosis of patients with this condition [3]. Approximately, knee osteoarthritis affected 10% of men and 13% of women aged 60 years or older in the USA [4]. The economic burden of knee osteoarthritis is substantial, with estimated lifetime care costs ranging from $12,400 to $16,000 [5]. The substantial economic burden of knee osteoarthritis underscores the need for cost-effective and sustainable therapeutic options. In this context, collagen peptides have emerged as a promising intervention due to their availability and potential benefits in promoting joint health. By addressing the pain and functional limitations associated with knee osteoarthritis, collagen peptides have the potential to improve the overall well-being of affected individuals while potentially reducing the financial strain associated with long-term care. Understanding the efficacy and safety of collagen peptides in the management of knee osteoarthritis can contribute valuable insights to optimize treatment strategies and provide relief to those impacted by this prevalent condition.

Collagen peptides are substances derived from hydrolyzed collagen and are also known as collagen hydrolysate. They are the basic building blocks that form the triple helix structure of collagen proteins, a prominent protein component of various connective tissues including skin, bones, tendons, and ligaments [6]. Collagen peptides are primarily composed of three amino acids: proline, hydroxyproline, and glycine, and can be extracted from bovine hides, fish scales, or chicken skins [7]. Collagen peptides are believed to improve overall health and appearance via stimulation of new collagen production in skin, maintaining its elasticity and firmness [8]. As a result, collagen peptides are often incorporated into cosmetic formulations, including creams, lotions and oral supplements that aim to reduce wrinkles and improve skin hydration [9]. Furthermore, Collagen peptides are believed to reduce joint pain and inflammation, enhance mobility, and support cartilage regeneration. However, the efficacy and potential benefits of collagen peptides in various applications remain a subject of ongoing scientific research and debate. Further studies are needed to better understand the effects and safety of collagen peptides in the management of knee osteoarthritis and other conditions [10].

Previous studies have reported the potential pain-relieving effects of collagen peptide or hydrolyzed collagen in people diagnosed with knee osteoarthritis [11, 12]. However, there is no meta-analysis or systematic review to evaluate the efficacy and safety of collagen peptides in this patient population. Therefore, to assess the efficacy of collagen peptide in relieving pain and reducing incidence of adverse events in patients with knee osteoarthritis, we conducted a meta-analysis of relevant literature.

## Methods

### Research protocol and search question

The study was completed based on the guidelines of the Preferred Reporting Items for Systematic Reviews and Meta-Analyses (PRISMA) statement. The protocol of this systematic review and meta-analysis study has been registered in PROSPERO (CRD42023429790). The target of this study was to evaluate the efficacy of collagen peptide or collagen hydrolysate in pain reduction and to assess potential risks compared to placebo in patients with osteoarthritis of the knee. PICO were defined as patients with knee OA (P), using collagen peptide or collagen hydrolysate (I), placebo (C), and pain score or adverse effect (O).

### Eligibility criteria and primary outcome

#### Types of studies

The included studies must be a randomized controlled trial (RCT). The study outcomes must include pain scores, such as visual analog scale (VAS), or adverse events. Trials were excluded if they were (1) single-arm follow-up studies, (2) case series, case reports, basic science experiments, reviews, or non-human studies, (3) conference abstracts, and (4) non-English articles.

#### Types of participants

The participants in the study must be diagnosed with knee osteoarthritis with either Kellgren-Lawrence grade I to III or functional class I to III.

#### Types of interventions

For a study to be included, its intervention must include hydrolyzed collagen as part of the intervention, and a matching amount of placebo as control. The substance used as placebo in the included studies involve lactose, maltodextrin, glucosamine sulfate, or a combination of maltodextrin, xanthan gum, and yeast extract.

### Search strategy and study selection

We comprehensively searched the following databases: PubMed, Scopus, EMBASE, Web of Science, and The Cochrane Library on May 27th, 2023. We used the Boolean algebra to explore the relevant keywords, and the search strategy was offered in Additional file 1. Moreover, the reference lists of the identified studies were screened to ensure a comprehensive search. Three individual reviewers (CRL, KYH, HC) assessed the eligibility of articles based on their titles and abstracts. The same reviewers then carried out an in-depth evaluation of the full-text articles to make the final decision on inclusion. Any disagreements between reviewers were resolved through a process of discussion and consensus.

### Data collection and quality assessment

Relevant data from the included trials were extracted by three independent reviewers (CRL, KYH, PAT). The extracted data included various study characteristics, including author details, year of publication, study location, data source, study design, sample size, patient age, inclusion criteria used in each study, and specific definitions for each treatment. Besides, the reviewers carefully documented outcomes of interest, such as pain scales (VAS) and the occurrence of adverse events. The efficacy of collagen peptides should be compared to the placebo group and only evaluated by VAS score on the 100-mm scale after intervention at the endpoint. The safety of collagen peptides was defined as any adverse events after administration at the endpoint of those studies. Two reviewers (CRL, PAT) assessed the risk of bias in the included studies and the quality of evidence for the study outcomes. The Cochrane ROB 2.0 framework [13] was used to evaluate the risk of bias, while the Grading of Recommendations, Assessment, Development, and Evaluations (GRADE) system [14] was used to assess the quality of evidence. Any disagreements between reviewers were resolved through discussion and consensus.

### Statistical analysis and quantitative data synthesis

A pairwise meta-analysis was performed to evaluate and compare the efficacy and safety of collagen peptides in people with knee osteoarthritis. We used standardized mean differences (SMDs) to assess the mean difference (MD) of analgesic effect of collagen peptides and odds ratio (OR) to examine the risk of adverse events of collagen peptides in knee osteoarthritis patients. The statistical heterogeneity of the results was assessed by categorizing the I^2^ values into different ranges. I^2^ values of 25% to 50%, 51% to 75%, and 76% to 100% were considered to indicate low, moderate, and high levels of statistical heterogeneity, respectively [15]. Given the expected clinical heterogeneity among the studies included in this meta-analysis, we used a random-effects model to estimate the aggregated results. A p-value of less than 0.05 was set as the threshold for statistical significance for all analyses. RevMan 5.4.1 was used to analyze the data.

## Results

### Literature search and selection process

**Fig. 1:**
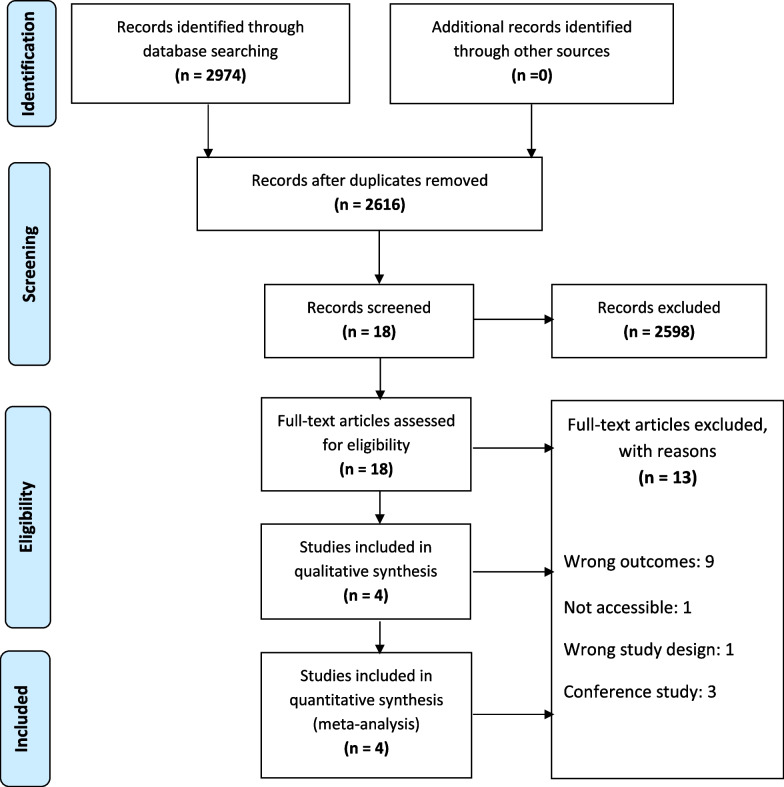
Flow of identification, screening, eligibility, and inclusion

After an extensive search of multiple databases, a total of 2974 records were identified. A strict screening process of titles and abstracts excluded duplicate and unrelated studies, resulting in 18 full-text articles being assessed for suitability. Four trials were eventually included in this meta-analysis, which comprised a total sample size of 507 patients diagnosed with knee osteoarthritis. (Fig. 1).

### Study characteristics

Table 1 summarizes the study characteristics of the included studies. The four studies were carried out in different countries, namely Ecuador (n = 207) [12], Taiwan (n = 113) [11], China (n = 94) [16], and the Czech Republic (n = 93) [17]. The interventions used in all trials were collagen peptides or hydrolyzed collagen, and each study was published as a full article. One study had two different intervention groups, hydrolyzed collagen type II and a combination of chicken essence and hydrolyzed collagen type II. [11]. All four studies used VAS to assess the analgesic effect of collagen peptides [11, 12, 16, 17]. One of the studies used VAS to rate pain intensity on several dimensions. These dimensions included current pain status, typical and average pain experiences, pain intensity at its peak, and pain intensity at its lowest point [17]. Table 2 reports the information on the formulation, source, and components used in the included studies.

**Table 1:** Study characteristics of the included studies

Study	Design	Location	Drug type	Inclusion criteria	Experimental group 1	Experimental group 2	Control group	Age (yrs)	Sex (M/F)	Outcome	Follow-up
Benito-Ruiz P 2009	Randomized, double-blind, controlled multicentre trial	Ecuador	Oral	Primary knee osteoarthritis (Kellgren-Lawrence grade I-III)	10 g hydrolyzed collagen for once-daily administration, 111 participants	NA	A matching amount of placebo (lactose) for once-daily administration, 96 participants	59	15/192	VAS (100 scale), WOMAC, SF-36 score	Baseline, 3 months, 5 months
Chen CC 2023	Randomized, double-blind, four-arm, pilot study	Taiwan	Oral	Mild to moderate knee osteoarthritis ( Kellgren–Lawrence grade I to III)	Hydrolyzed collagen type II, 38 participants	Essence of chicken + hydrolyzed collagen type II, 37 participants	6.8 g maltodextrin, 0.007 g xanthan gum, and 0.43 g yeast extract, 38 participants	45–75 years	17/96	VAS pain score, WOMAC score, fat-free mass, grip strength, SF-36	WOMAC score at 8, 16, and 24 weeks; VAS pain score at 7 and 14 days; grip strength, FFM at 24 weeks
JX Jiang 2014	Prospective,single-centre,randomized,doulble-blind,placebo-controlled trial	China	Oral	Knee osteoarthritis (Kellgren- Lawrence score of 0-I to III)	Peptan B 2000 of bovine origin,8 g per day for 6 months, 46 paticipants	NA	Maltodextrin,8 g per day for 6 months,48 participants	40–70	0/94	WOMAC and the Lysholm scoring system	3 months,6 months
Trč T 2011	Prospective, multicentre, randomized, parallel, double-blind study	Czech Republic	Oral	Knee osteoarthritis (functional class I, II, or III)	10 g enzymatic hydrolyzed collagen once daily for 90 consecutive day, 47 participants	NA	1.5 g glucosamine sulfate once daily for 90 consecutive day, 46 participants	40 years or older	NA	VAS (100 scale), WOMAC, SF-36 score, self-assessment, the investigator’s overall opinion, and reduction of the use of rescue medication	− 7, 0, 15, 30, 60, and 90 days

**Table 2:** Patented formulations, botanical or chemical of medications from the included studies

Study	Formulation	Source	Component	Quality control reported? (Y/N)	Chemical analysis reported? (Y/N)
Benito 2009	Colnatur (Protein SA, Girona, Spain), a powdered hydrolyzed natural collagen with a mean molecular weight of 3,500 Da; 10 g hydrolyzed collagen for once-daily administration dissolved in a liquid of the patient’s choice	Colnatur (Protein SA, Girona, Spain)	natural hydrolyzed collagen	N	N
Chen CC 2023	Essence of Chicken-hydrolyzed collagen type II doses: 2.0 g of hydrolyzed collagen type II collagen and 5.81 g of Essence of Chicken with proteins and peptides/hydrolyzed collagen type II doses: 2.0 g of hydrolyzed collagen type II collagen	Suntory Beverage and Food Asia (Changhua Taiwan, Good Hygiene Practice certified)	Hydrolyzed collagen type II: derived from chicken sternal cartilage; Essence of Chicken: extracted from chicken meat	N	Y
JX jiang 2014	Hydrolyzed collagen bovine 100%	Peptan B 2000,Rousselot	Food grade Bovine Collagen Peptides	Y	N
Trč T 2011	10 g enzymatic hydrolyzed collagen	Colatech® (trade name, uncertain company)	Enzymatic hydrolyzed collagen	N	N

### Methodological quality and assessment of risk of bias

**Fig. 2:**
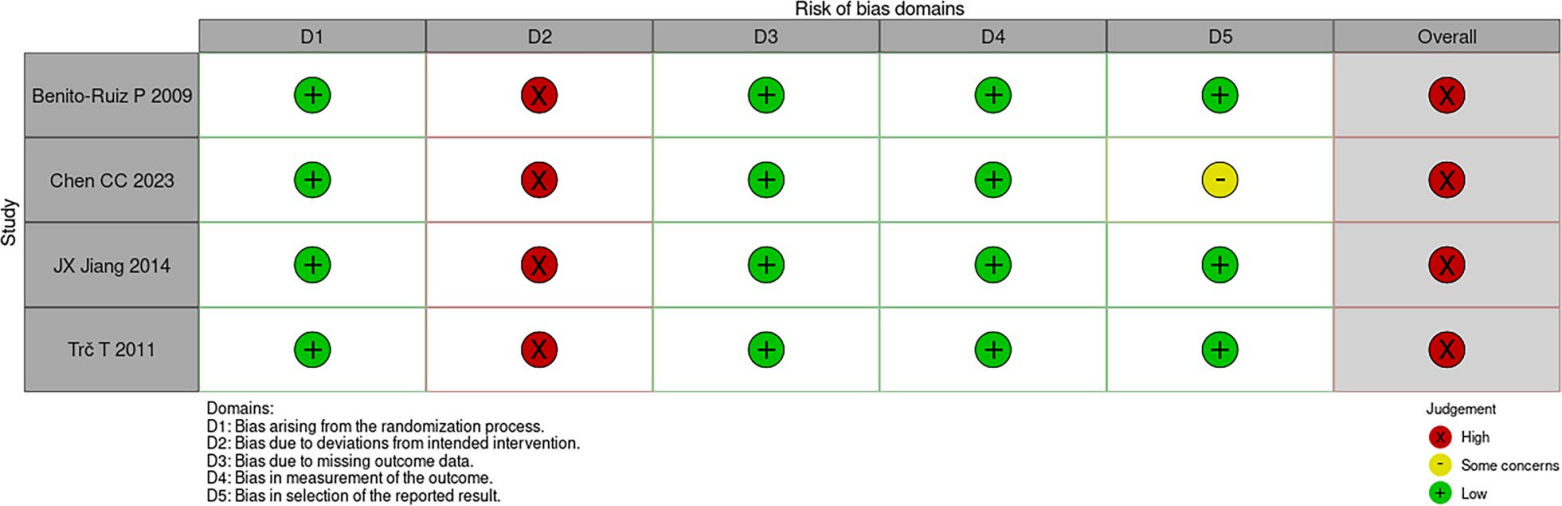
ROB2, risk of bias assessment of the included studies, and the summary of domains

**Fig. 3:**
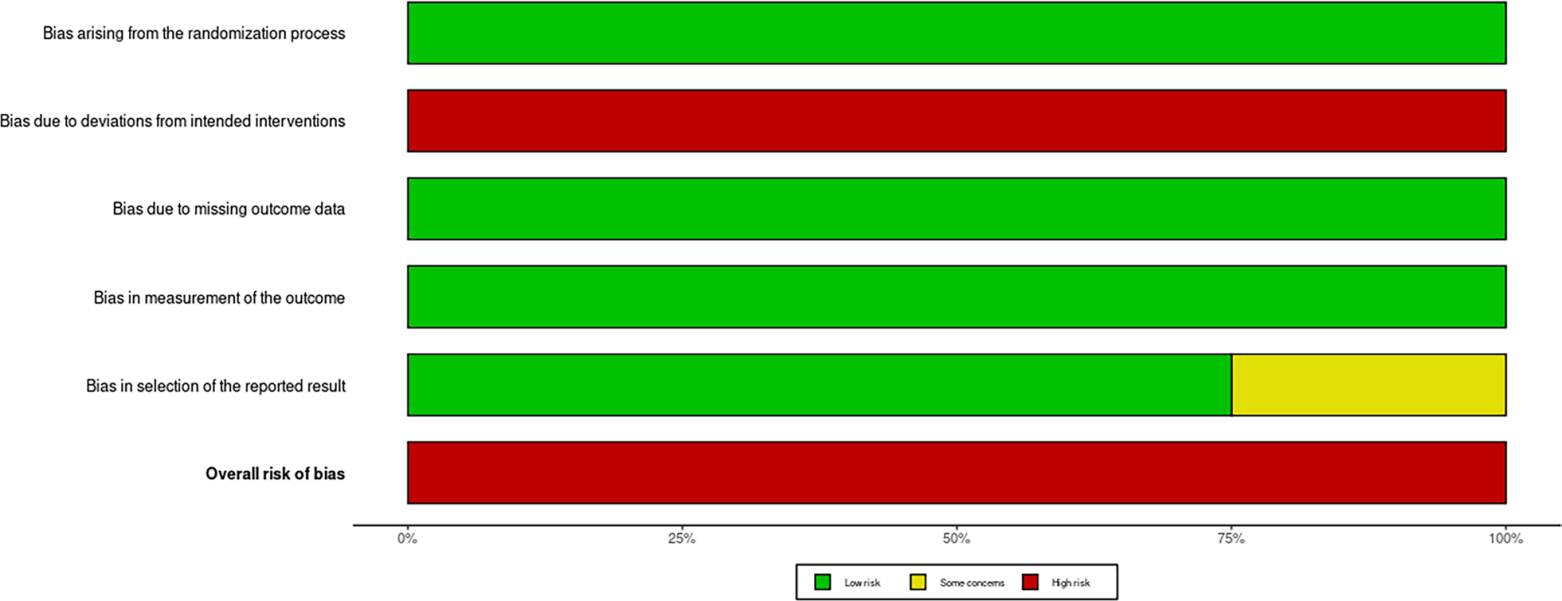
The summary of domains of the risk of bias

As shown in Figs. 2 and 3, all four studies included in this analysis were assessed as having an overall high risk of bias using the ROB 2.0 tool [13]. Specifically, in terms of bias due to deviations from the intended interventions, none of the four studies provided an adequate analysis to estimate the effect of non-adherence [11, 12, 16, 17]. Furthermore, in terms of risk of bias in the selection of reported outcomes, one study did not present all relevant data in precise numerical values but relied on graphical representations [11].

### Pain

**Fig. 4:**
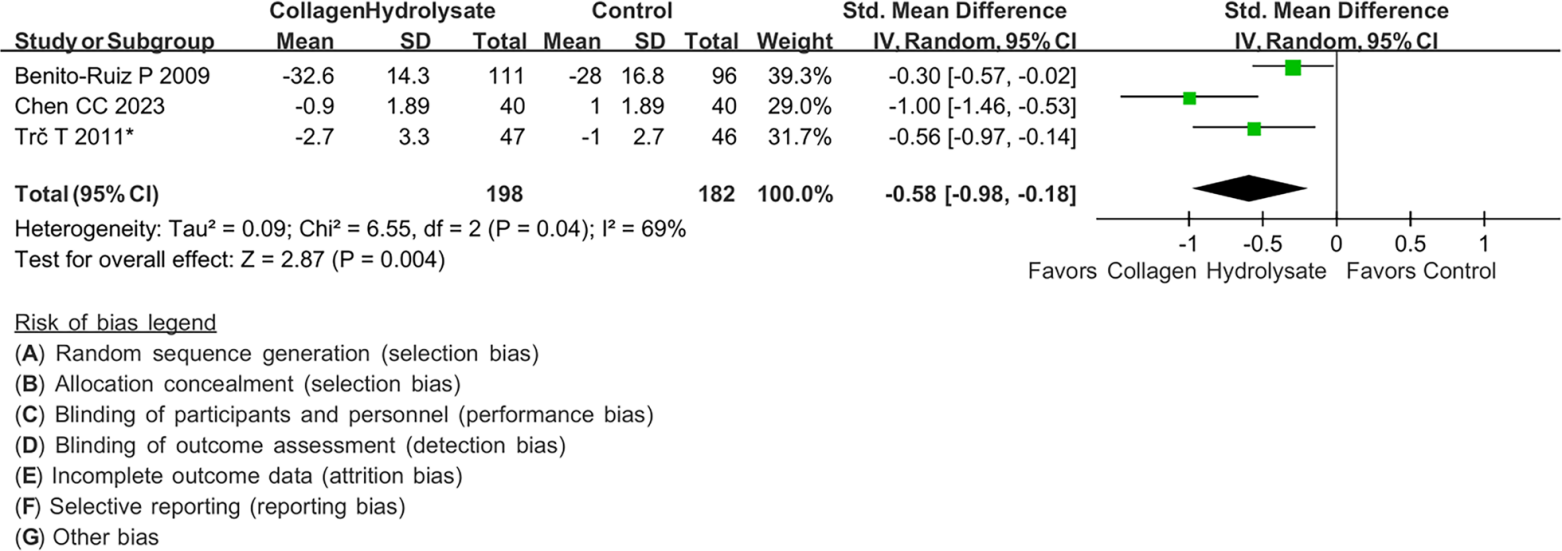
Forest plot demonstrating overall pain scores comparing between collagen peptides and placebo. Better pain control is shown if favor collagen peptides or placebo

A total of three studies with 375 patients with knee osteoarthritis were included in the evaluation of the analgesic efficacy of collagen peptides. In Fig. 4, our meta-analysis reported a statistically significant difference in pain control in patients with knee osteoarthritis when comparing the collagen peptide and placebo groups (SMD: − 0.58; 95% CI − 0.98, − 0.18, p = 0.004; I2: 68%; quality of evidence: moderate).

### Adverse event

**Fig. 5:**
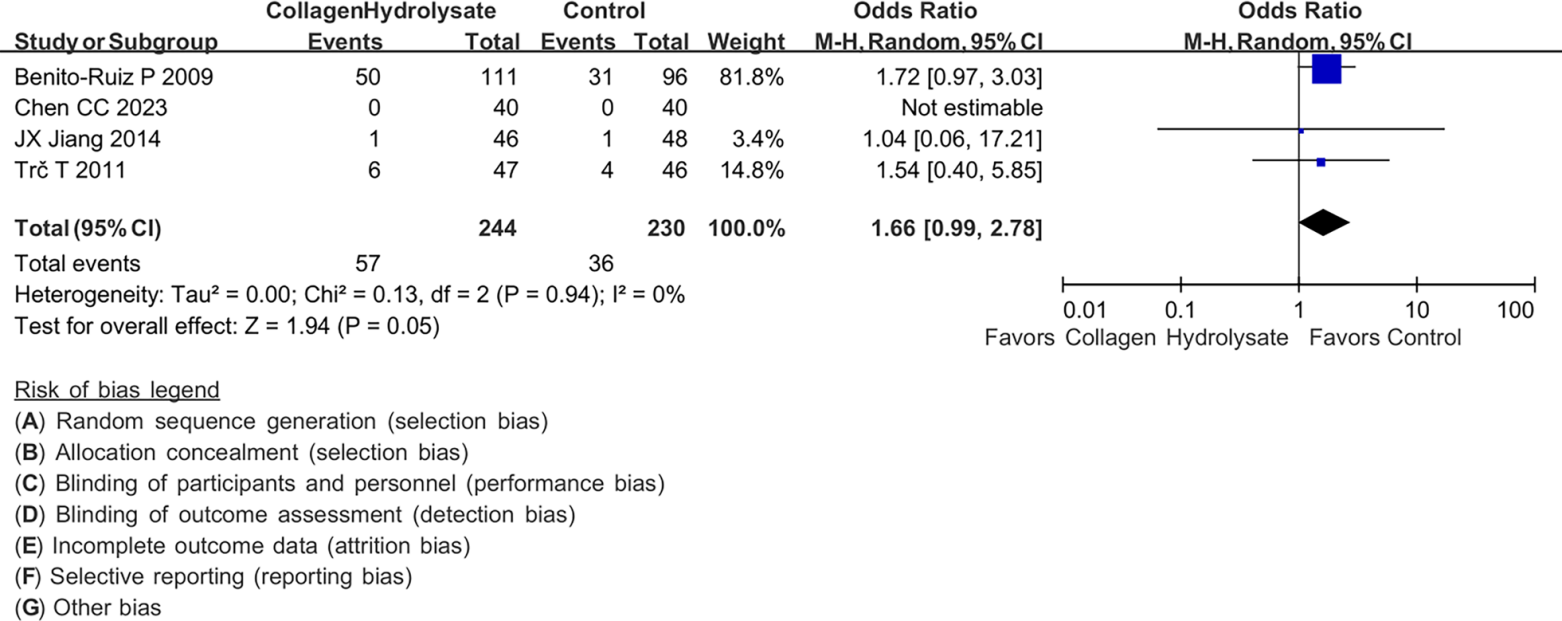
Forest plot demonstrating overall adverse events between collagen peptides and placebo. Lower adverse event is shown if favor collagen peptides or placebo

A total of four studies with 507 patients with knee osteoarthritis were included in the evaluation of the risk of adverse events of collagen peptides. The adverse events reported in the studies include migraine headache, gastrointestinal morbidities, respiratory infections, septic arthritis, and lateral thigh pain. The difference in adverse events between the collagen peptide and placebo groups is shown in Fig. 5. Our meta-analysis showed no significant difference in the risk of adverse events between the collagen peptide and placebo groups in patients with knee osteoarthritis (OR 1.66; 95% CI 0.99, 2.78, p = 0.05; I^2^:0%; quality of evidence: very low).

### Post hoc analysis

**Fig. 6:**
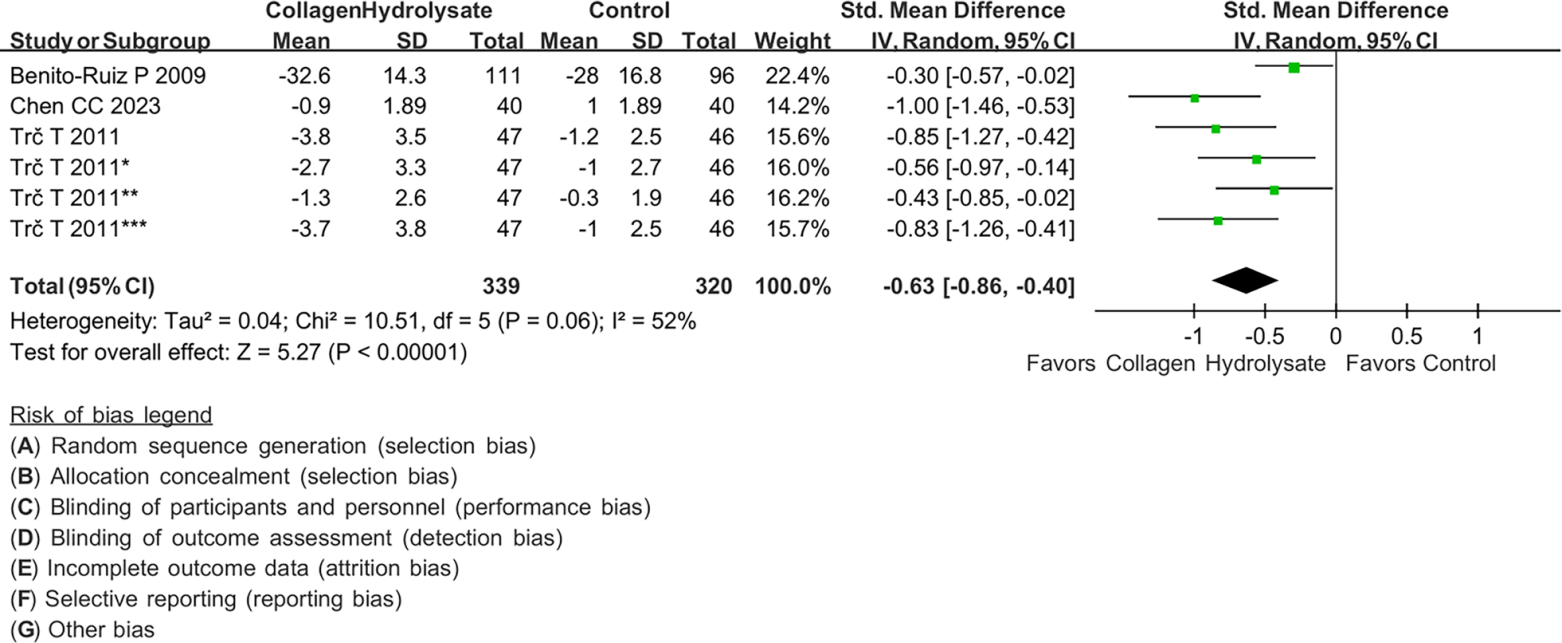
Post hoc analysis of pain scores including data in all different conditions in Trč 2011 between collagen peptide and placebo. Better pain control is shown if favor collagen peptides or placebo. Trč 2011: VAS measured in pain right now. Trč 2011*: VAS measured of typical or average pain Trč 2011**: VAS measured of pain level at its best. Trč 2011***: VAS measured of pain level at its worst

The study conducted by Trč and BohmováIn reported an analgesic effect in 4 different conditions, including VAS in pain right now, typical or average pain, pain level at its best and pain level at its worst [17]. Thus, in our additional analysis, we extracted the data of the other groups from the study conducted by Trč and Bohmová [17]. The difference in analgesic effect between the collagen peptide and placebo groups is shown in Fig. 6. Our meta-analysis revealed a statistically significant difference in pain control in patients with knee osteoarthritis when comparing the collagen peptide and placebo groups (SMD: − 0.63; 95% CI − 0.86, − 0.39, p < 0.00001; I2: 52%; quality of evidence: moderate).

## Discussion

Our study yielded promising findings regarding the potential clinical benefits of collagen peptides in providing pain relief for individuals with knee osteoarthritis. The analysis showed a significant difference in pain relief between the collagen peptide group and the placebo group. Importantly, the study also revealed that the risk of adverse events did not significantly differ between the two groups. The most commonly reported adverse events associated with collagen peptide administration were gastrointestinal disorders, migraines, and collagen peptide-related infections including respiratory infection and septic arthritis. These results suggest that collagen peptides may offer a safe and effective therapeutic option for managing knee osteoarthritis symptoms.

With the trend of the global population aging, the prevalence of osteoarthritis patients rose, highlighting the urgent need for effective osteoarthritis treatment and prevention strategies. Owing to the potential adverse effects associated with the use of analgesics and anti-inflammatory drugs for the treatment of osteoarthritis, it is necessary to explore safe therapeutic ingredients to replace or minimize reliance on currently used treatment modalities [18, 19]. Collagen derivatives, including collagen hydrolysate, undenatured collagen, and gelatine, are candidates for use as disease-modifying drugs for osteoarthritis [20]. In addition, a previous study which contained more than 60 studies (in vitro, in vivo, clinics and on bioavailability) exploring the impact of collagen peptides on cartilage damage, joint erosions, and joint pain reported consistent intake of collagen peptides have benefit in prevention and relief of joint discomfort, reducing bone density loss, and slowing skin aging process [21].

Collagen peptide products have long been used in pharmaceuticals, biomaterials, and foods [7]. Our meta-analysis revealed an analgesic effect of collagen peptide in patients with knee osteoarthritis compared to the placebo groups. Multiple studies demonstrated possible cellular mechanisms of the beneficial effects of collagen peptides on alleviating pain and improving joint condition in knee osteoarthritis. These mechanisms include the anti-inflammatory and antioxidant capacities of collagen peptides, and its ability to stimulate collagen synthesis and promote bone formation [22–24]. In vitro and in vivo studies showed a reduction of pro-inflammatory cytokines, including IL-1β, IL-6, and TNF-α after collagen peptide administration [22, 25]. Collagen peptide also displays antioxidant activities measured by oxygen radical absorbance capacity and radical scavenging assay [24]. Furthermore, evidence has shown that orally administered collagen hydrolysate stimulates a significant increase in type II collagen synthesis by chondrocytes [26]. Animal studies demonstrated the efficacy of gelatin to increase type I collagen and glycosaminoglycan content as well as bone mineral density in the femur of rats [27]. Bovine collagen hydrolysate was shown to stimulate osteoblast differentiation and mineralized bone matrix formation through increased runt-related transcription factor 2 (Runx2) expression and activity [23], and may serve as an effective supplement for preventing bone loss by significantly enhancing the organic substance content of bone [28]. The promotion of bone formation could be further explained by the downregulation of the aforementioned pro-inflammatory molecules, because these cytokines are responsible for the upregulation of receptor activator for nuclear factor kappa-B ligand (RANKL) for osteoclast recruitment, which may lead to bone loss [25]. Current scientific research indicates that consistent consumption of collagen peptides has been associated with a reduction in joint pain and bone density loss [21, 22, 29]. The underlying mechanisms through which collagen peptides exert these beneficial effects may involve their ability to diminish proinflammatory molecules, enhance collagen synthesis, and facilitate bone formation.

Our results showed no significant difference in the risk of adverse events between the collagen peptide and placebo groups in patients with knee osteoarthritis. However, not all of the studies included in our research [11, 12, 16, 17] specified the adverse events. In the study by Benito-Ruiz P et al., the most common adverse event of collagen peptide was gastrointestinal disorders (n = 29), followed by migraine headache (n = 14) and respiratory infection (n = 10). However, none of the adverse events were obviously associated with the treatment [12]. A previous RCT of collagen peptides also reported gastrointestinal symptoms as the most common adverse events, which is compatible with our findings. These gastrointestinal symptoms, including vomiting and diarrhea, were of mild to moderate severity, and were cured by medical interventions [10]. Other less common adverse events reported include septic arthritis and allergic peripheral edema [10, 16]. A previous RCT demonstrated a significant improvement in liver function indicators (serum glutamic oxaloacetic transaminase and serum glutamic pyruvic transaminase) and blood urea nitrogen in the collagen peptide group compared to the placebo, indicating the safety of collagen peptide use for knee osteoarthritis [16]. In another systematic review, none of the involved studies reported side effects of collagen peptide [6], which was similar to our findings. Overall, collagen peptide is safe with a high level of tolerance, making it a potential supplement or medication for long-term use in knee osteoarthritis [21].

Our study provides compelling evidence supporting the therapeutic potential of collagen peptides in the treatment of knee osteoarthritis. It is important to note that a previous meta-analysis by Van Vijven et al. reported limited efficacy of collagen derivatives in osteoarthritis [20]. However, the previous meta-analysis included a broader range of collagen derivatives, namely gelatin, undenatured collagen type II, and collagen peptides. In contrast, our study specifically concentrated on evaluating the therapeutic effects of collagen peptides alone. This difference in the composition of collagen derivatives examined could potentially contribute to variations in the observed outcomes. Moreover, the previous meta-analysis encompassed osteoarthritis of various organs, while our research exclusively focused on knee osteoarthritis. Osteoarthritis can affect different joints and organs throughout the body, and the pathophysiological processes and responses to treatment may vary across these different locations. By narrowing our scope to knee osteoarthritis, we aimed to provide a more targeted analysis of the specific benefits and effects of collagen peptides in this particular context. Therefore, the differences in the types of collagen derivatives examined and the focus on knee osteoarthritis specifically are key factors that likely contribute to the contrasting findings between the previous meta-analysis and our study. It is crucial to consider these distinctions when interpreting the results and implications of each study.

Nevertheless, our study is not without limitations. Firstly, the inclusion of only four trials in this meta-analysis resulted in a relatively small sample size, which may impact the generalizability of our findings. In addition, the studies included in our analysis had variations in their design, including differences in the dosage and components of the collagen peptides used, which may have influenced the observed analgesic effects. It is important to consider the clinical heterogeneity when interpreting the results, which is why we employed the GRADE system and a random-effect model for our analysis. Moreover, one study reported pain scores using the VAS across multiple dimensions, which may have disproportionately influenced the overall results. To address this concern, a post hoc analysis was performed in our study. Table 3 recapitulates the results of the GRADE assessment [14] for the included studies.

**Table 3:** GRADE (Grading of Recommendations, Assessment, Development and Evaluations) criteria for assessing quality of evidence

Outcome	Number of studies	Number of participants	Risk of bias	Imprecision	Inconsistency	Indirectness	Publication bias	Relative effect (95% confidence interval)	Confidence in effect estimate (Grade)
Pain	3	375	Serious	Not serious	Moderate	Not serious	Not serious	− 0.63 (95% CI − 0.86, − 0.39)	Moderate
Adverse effect	4	507	Serious	Not serious	Not serious	Not serious	Serious	1.66 (95% CI 0.99, 2.78)	Very low

It is worth highlighting that our meta-analysis represents the first systematic review of randomized controlled trials (RCTs) investigating the clinical benefits of collagen peptides for pain relief in patients with knee osteoarthritis. However, further well-designed RCTs are warranted in future research to validate and strengthen our findings. Continued investigation will enhance the understanding of the efficacy and safety of collagen peptides as a therapeutic option for knee osteoarthritis, ultimately providing more robust evidence for clinical decision-making.

## Conclusion

In conclusion, our systematic review and meta-analysis provides compelling evidence of significant pain reduction in knee osteoarthritis patients who received collagen peptides compared to those who received a placebo. Furthermore, we observed no significant difference in the risk of adverse events between the collagen peptide and placebo groups in this patient population. However, the certainty and evidence of our results is limited due to potential bias, small sample sizes, and inconsistencies within the included trials. To validate and reinforce our conclusions, it is imperative that further well-designed RCTs be conducted. These future studies will enhance the reliability of the evidence and provide a more comprehensive understanding of the potential benefits and safety profile of collagen peptides in knee osteoarthritis management.

### Supplementary Information

**Additional file 1.** **Appendix 1.** Search strategies.

## Data Availability

All the data in this study was reported in the article. For further information, please contact the corresponding author.
