# Supplementary material for: Analgesic efficacy of collagen peptide in knee osteoarthritis: a meta-analysis of randomized controlled trials
Source: J Orthop Surg Res. 2023 Sep 16;18:694. doi: 10.1186/s13018-023-04182-w (PMC10505327; doi:10.1186/s13018-023-04182-w)
Supplement: Supplementary file 1 — Additional file 1. Appendix 1. Search strategies. [file 13018_2023_4182_MOESM1_ESM.docx]

**Appendix 1. Search strategies**

**Pubmed**

1. Exp collagen hydrolysate/
2. Exp collagen peptide/
3. Exp collagen peptides/
4. Or/1-3
5. Exp osteoarthritis/
6. Exp knee/
7. 5 And 6
8. randomized.tiab.
9. placebo.tiab.
10. randomly.tiab.
11. trial.tiab.
12. groups.tiab.
13. comparative.tiab.
14. cohort.tiab.
15. randomised.tiab.
16. Or/8-15
17. 4 AND 7 AND 16

**Embase**

1. Exp collagen hydrolysate/
2. Exp collagen peptide/
3. Exp collagen peptides/
4. Or/1-3
5. Exp osteoarthritis/
6. Exp knee/
7. 5 And 6
8. randomized.ti.ab.kw.
9. placebo.ti.ab.kw.
10. randomly.ti.ab.kw.
11. trial.ti.ab.kw.
12. groups.ti.ab.kw.
13. comparative.ti.ab.kw.
14. cohort.ti.ab.kw.
15. randomised.ti.ab.kw.
16. Or/8-15
17. 4 AND 7 AND 16

**Scopus**

1. Exp collagen hydrolysate/
2. Exp collagen peptide/
3. Exp collagen peptides/
4. Or/1-3
5. Exp osteoarthritis/
6. Exp knee/
7. 5 And 6
8. randomized.ti.ab.kw.
9. placebo.ti.ab.kw.
10. randomly.ti.ab.kw.
11. trial.ti.ab.kw.
12. groups.ti.ab.kw.
13. comparative.ti.ab.kw.
14. cohort.ti.ab.kw.
15. randomised.ti.ab.kw.
16. Or/8-15
17. 4 AND 7 AND 16

**The Cochrane Library**

1. Exp collagen hydrolysate/
2. Exp collagen peptide/
3. Exp collagen peptides/
4. Or/1-3
5. Exp osteoarthritis/
6. Exp knee/
7. 5 And 6
8. randomized.ti.ab.kw.
9. placebo.ti.ab.kw.
10. randomly.ti.ab.kw.
11. trial.ti.ab.kw.
12. groups.ti.ab.kw.
13. comparative.ti.ab.kw.
14. cohort.ti.ab.kw.
15. randomised.ti.ab.kw.
16. Or/8-15
17. 4 AND 7 AND 16

**Web of science**

1. Exp collagen hydrolysate/
2. Exp collagen peptide/
3. Exp collagen peptides/
4. Or/1-3
5. Exp osteoarthritis/
6. Exp knee/
7. 5 And 6
8. Exp randomized/
9. Exp placebo/
10. Exp randomly/
11. Exp trial/
12. Exp groups/
13. Exp comparative/
14. Exp cohort/
15. Exp randomised/
16. Or/8-15
17. 4 AND 7 AND 16
